# Unique miRNome and transcriptome profiles underlie microvascular heterogeneity in mouse kidney

**DOI:** 10.1152/ajprenal.00005.2023

**Published:** 2023-07-06

**Authors:** Matthijs Luxen, Peter J. Zwiers, Femke Meester, Rianne M. Jongman, Timara Kuiper, Jill Moser, Marianne Pultar, Susanna Skalicky, Andreas B. Diendorfer, Matthias Hackl, Matijs van Meurs, Grietje Molema

**Affiliations:** ^1^Department of Pathology and Medical Biology, Medical Biology Section, University Medical Center Groningen, University of Groningen, Groningen, The Netherlands; ^2^Department of Critical Care, University Medical Center Groningen, University of Groningen, Groningen, The Netherlands; ^3^Department of Anaesthesiology, University Medical Center Groningen, University of Groningen, Groningen, The Netherlands; ^4^TAmiRNA GmbH, Vienna, Austria

**Keywords:** endothelial cells, microvascular heterogeneity, miRNome, renal microvascular compartments, transcriptome

## Abstract

Endothelial cells in blood vessels in the kidney exert different functions depending on the (micro)vascular bed they are located in. The present study aimed to investigate microRNA and mRNA transcription patterns that underlie these differences. We zoomed in on microvascular compartments in the mouse renal cortex by laser microdissecting the microvessels prior to small RNA- and RNA-sequencing analyses. By these means, we characterized microRNA and mRNA transcription profiles of arterioles, glomeruli, peritubular capillaries, and postcapillary venules. Quantitative RT-PCR, in situ hybridization, and immunohistochemistry were used to validate sequencing results. Unique microRNA and mRNA transcription profiles were found in all microvascular compartments, with dedicated marker microRNAs and mRNAs showing enriched transcription in a single microvascular compartment. In situ hybridization validated the localization of microRNAs mmu-miR-140-3p in arterioles, mmu-miR-322-3p in glomeruli, and mmu-miR-451a in postcapillary venules. Immunohistochemical staining showed that von Willebrand factor protein was mainly expressed in arterioles and postcapillary venules, whereas GABRB1 expression was enriched in glomeruli, and IGF1 was enriched in postcapillary venules. More than 550 compartment-specific microRNA-mRNA interaction pairs were identified that carry functional implications for microvascular behavior. In conclusion, our study identified unique microRNA and mRNA transcription patterns in microvascular compartments of the mouse kidney cortex that underlie microvascular heterogeneity. These patterns provide important molecular information for future studies into differential microvascular engagement in health and disease.

**NEW & NOTEWORTHY** Renal endothelial cells display a high level of heterogeneity depending on the (micro)vascular bed they reside in. The molecular basis contributing to these differences is poorly understood yet of high importance to increase understanding of microvascular engagement in the kidney in health and disease. This report describes m(icro)RNA expression profiles of microvascular beds in the mouse renal cortex and uncovers microvascular compartment-specific m(icro)RNAs and miRNA-mRNA pairs, thereby revealing important molecular mechanisms underlying renal microvascular heterogeneity.

## Graphical abstract

**Figure:**
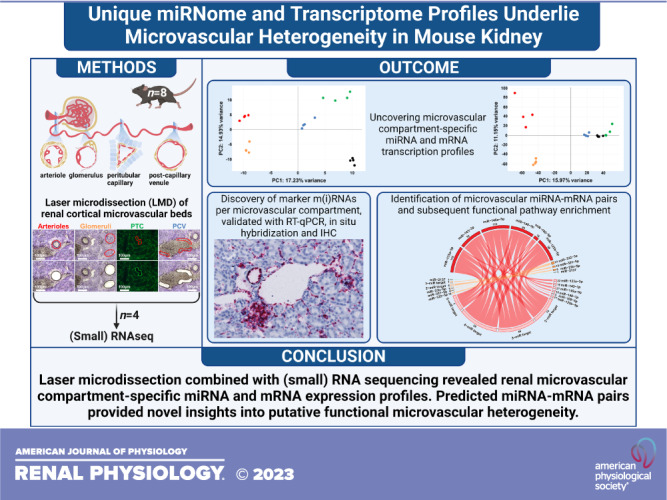

## INTRODUCTION

The endothelium forms the inner lining of blood vessels and is present in every organ in the body. Endothelial cells (ECs) play crucial roles in various processes, including control of vasomotor tone, coagulation, selective barrier function, and recruitment of circulating leukocytes. A unique endothelial characteristic is a highly heterogeneous contribution to these processes depending on the microvascular bed in which they reside. In the kidney cortex, four different microvascular beds containing different endothelial subsets can be identified: arterioles, glomeruli, peritubular capillaries, and postcapillary venules, which all play unique functional roles in the kidney (1). These functional differences are likely accompanied by molecular differences, such as cell surface receptor expression being exclusive to specific microvascular beds or intracellular signal transduction pathways taking different routes due to differential expression of adapter molecules (1, 2). Within the renal microvasculature, a well-known example of this heterogeneity is the high expression of vascular endothelial growth factor receptor 2 on the glomerular endothelium compared with other microvascular beds (3). Unraveling the molecular basis of endothelial heterogeneity could help explain why ECs in some microvascular beds become dysfunctional, e.g., in response to proinflammatory stimuli, whereas others do not (4–6). This knowledge could facilitate pharmacological targeting of ECs in specific microvascular beds to prevent dysfunctional responses from taking place (7, 8).

MicroRNAs (miRNAs) represent potential, albeit understudied, contributors to endothelial heterogeneity. miRNAs are small noncoding RNA molecules that can interact with target mRNA sequences, thereby inhibiting their translation into proteins. Although miR-126 is commonly known to be endothelium enriched (9), little is known about which miRNAs are transcribed in different endothelial subsets and whether they functionally control the unique properties of different microvascular beds. The contribution of differential miRNA transcription patterns to diverse biological effects is illustrated by the fact that high relative transcription of miR-126 in glomerular ECs is associated with inhibited expression of vascular cell adhesion molecule 1 protein in response to proinflammatory stimuli (6). Increased understanding of miRNA involvement in deleterious endothelial processes is also of interest from a therapeutic perspective, as miRNAs have therapeutic potential to attenuate endothelial dysfunction in vivo (9).

Various examples of heterogeneous mRNA and protein expression profiles in ECs have been reported (1, 10–12), and, in recent years, single-cell RNA sequencing (scRNA-seq) further enabled characterization of cellular heterogeneity and the discovery of numerous endothelial subsets (13–17). Although a main advantage of using scRNA-seq to study ECs is that relatively pure cell (sub)populations are obtained, ECs are notorious for rapidly adjusting their transcriptome in response to altered circumstances, for instance, when being enzymatically released from their in vivo environment or when brought into culture (18). This feature makes it particularly challenging when using scRNA-seq to discern which genes are expressed in vivo and which genes are changed as a result of sample handling and enzymatic cell dissociation protocols. Moreover, the technological tools to identify miRNA transcription patterns at the single-cell level are still in their infancy (19).

The aim of the present study was to develop a workflow to combine laser microdissection (LMD) of different microvascular beds present in the mouse kidney cortex with small RNA-seq and RNA-seq readout technologies and to make a molecular map of microvascular miRNA and mRNA signatures. A major advantage of LMD compared with scRNA-seq platforms is that the experimental procedure does not change the molecular makeup of cells, and it has the additional benefit of preserving spatial information. Our approach enabled identification of miRNA and mRNA signatures of each microvascular compartment, which were validated with quantitative RT-PCR, in situ hybridization (ISH), and immunohistochemistry (IHC). We furthermore compared the molecular signatures with recently published scRNA-seq studies and linked identified microvascular miRNAs to their mRNA targets. The findings reported here provide novel insights into the technical feasibility of combining LMD with sequencing platforms and describe miRNA and mRNA profiles underlying microvascular heterogeneity in the kidney.

## METHODS

### Mice

Eight- to 12-wk-old C57BL/6 OlaHsd male mice (20–30 g, Envigo, Horst, the Netherlands) were maintained at 24°C with 12:12-h light-dark cycles with ad libitum access to food and water. Experimental procedures were approved by and performed in accordance with the University of Groningen ethical committee and National and European guidelines for animal care and use (No. 8116, AVD1050020184904).

Mice (*n* = 8) were anesthetized by isoflurane inhalation before euthanasia by cardiac puncture. All organs, including the kidney, were harvested and either snap frozen on liquid nitrogen and stored at −80°C until further analysis or fixed with a zinc-based fixative [3.2 mM calcium acetate, 27.3 mM zinc acetate, and 36.7 mM zinc chloride in 0.1 M Tris (pH 7.4), all from MilliporeSigma, Burlington, MA] for 24 h, embedded in paraffin, and stored at room temperature (20).

Sample blinding and randomization were not required in the current experimental setup, as all mice were healthy control animals.

### Laser Microdissection of Renal Microvascular Segments

To laser microdissect microvascular structures from healthy mouse kidneys (*n* = 8), 9-µm-thick cryosections from snap-frozen kidneys were put on MembraneSlides 1.0 PEN (D) (Zeiss, Oberkochen, Germany), fixed, stained, washed, and air-dried, as previously described (21). Per mouse, one slide was stained with Mayer’s hematoxylin (Sigma-Aldrich, St. Louis, MO) for the collection of arteriolar (5 × 10^5^ µm^2^), glomerular (1 × 10^6^ µm^2^), and venous (postcapillary venules (1 × 10^6^ µm^2^) microvascular segments. Another slide was stained with FITC-conjugated *Griffonia simplicifolia* lectin I isolectin B4 (Vector Laboratories, Newark, CA) for the collection of peritubular capillaries (5 × 10^5^ µm^2^). LMD was performed with a Leica LMD7000 microscope (Leica, Wetzlar, Germany) using Laser Microdissection System software (v. 8.2.3.7603, Leica), and dissected tissue was collected in 0.5-mL AdhesiveCap Opaque 500 tubes (Zeiss). Samples were stored at −80°C until further analysis.

### Total RNA Isolation

Total RNA from LMD samples and whole kidney sections was isolated using NucleoZOL lysis reagent with NucleoSpin RNA Set for NucleoZOL (both from Macherey-Nagel, Düren, Germany) according to the manufacturer’s protocols. Glycogen (Invitrogen, Waltham, MA) was added to the sample homogenate at a final concentration of 50 µg/mL before the sample was loaded onto the column. The RNA concentration and purity (optical density: 260/280) of whole kidney sections were determined with a NanoDrop ND-1000 UV-vis spectrophotometer (NanoDrop Technologies, Wilmington, DE).

### Small RNA Sequencing

Small RNA libraries were prepared with equal amounts of total RNA (100 ng) for whole kidney sections and with a fixed volume (8.5 µL) of total RNA for LMD samples with a RealSeq Biofluids library preparation kit (RealSeq Biosciences, Santa Cruz, CA) according to the manufacturer’s protocols (*n* = 4). The RNA integrity and RNA concentration of whole kidney samples were analyzed using Agilent Bioanalyzer RNA 6000 Nano Chip (Agilent Technologies, Santa Clara, CA) according to the manufacturer’s instructions. Adapter-ligated libraries of whole kidney samples were amplified with 18 cycles and LMD samples with 24 cycles using barcoded Illumina reverse primers in combination with the Illumina forward primer (Illumina, San Diego, CA). Three pools of 24 small RNA-seq libraries were prepared at equimolar amounts based on DNA1000 Bioanalyzer results (Agilent Technologies). Sequencing was performed on an Illumina HiSeq V4 (Illumina) with 50-bp single-end reads. Sequencing reads were adapter trimmed and filtered for low-quality reads (Q < 30). miRNA identification and annotation were performed using the miRNA NGS Discovery (miND) pipeline (22). Read counts were normalized to the total number of miRNA reads detected per sample to obtain the reads per million (RPM) for each miRNA and sample. Exploratory data analysis was performed using unsupervised cluster analysis, and differential expression analysis was performed using the statistical methods available under the edgeR package in R/Bioconductor (23).

### RNA Sequencing

RNA-seq libraries were prepared from RNA of whole kidney samples (100 ng) and LMD samples (5 µL) using QuantSeq 3′ mRNA Library kit FWD for Illumina (Lexogen, Vienna, Austria) according to the manufacturer’s protocol (*n* = 4). For LMD libraries, the protocol modifications for low-input samples were used. Whole kidney libraries were amplified with 18 PCR cycles and LMD sample libraries with 24 cycles. After purification, library yield was determined with DNA High Sensitivity analysis on a Bioanalyzer. A pool of 72 RNA-seq libraries was prepared at equimolar amounts, and sequencing was performed on a NovaSeq SP System (Illumina) with 100-bp single-end reads.

Overall quality of the next-generation sequencing data was evaluated automatically and manually with fastQC v. 0.11.8 and multiQC v. 1.7 (24, 25). Reads from all passing samples were adapter trimmed and quality filtered using bbduk from the bbmap package v. 38.69 and filtered for a minimum length of 17 nt and phred quality of 30 (26). Alignment steps were performed with STAR v. 2.7 using samtools v. 1.9 for indexing (27, 28), and reads were mapped against the genomic reference GRCm38.p6 provided by Ensembl (29). Assignment of features to the mapped reads was done with htseq-count v. 0.13 (30). Differential expression analysis with edgeR v. 3.30 used the quasi-likelihood negative binomial generalized log-linear model functions provided by the package (23). The independent filtering method of DESeq2 was adapted for use with edgeR to remove low abundant genes and thus optimize the false discovery rate correction (31).

### Sequencing Data Analysis

Enrichment of m(i)RNA transcription per microvascular compartment was determined by performing differential expression analysis of a single microvascular compartment versus the whole kidney.

To identify microvascular compartment-specific m(i)RNAs, differential expression analysis was performed for one microvascular compartment versus a mixture of the remaining three microvascular compartments. m(i)RNAs with the highest differential expression were selected as “marker m(i)RNAs,” provided all replicates exhibited RPM ≥ 10 for the studied microvascular compartment.

### Pathway Enrichment Analysis and miRNA-mRNA Pair Analysis

Gene ontology (GO) enrichment analysis was performed using the Bioconductor package topGO v. 2.50.0 (32). Enriched biological processes were identified using a Kolmogorov-Smirnov (KS) test by including mRNAs with a positive log fold change (FC) and false discovery rate < 0.05 based on differential expression results of RNA-seq data. The unadjusted *P* value was used as a rank weight in the KS test. No significantly enriched biological processes (KS *P* < 0.05) were identified for postcapillary venules.

Predicted targets for the previously identified marker miRNAs were derived using the R package multiMiR v. 1.20.0 (33). The union intersection of the top 90% predicted targets of eight databases was used, i.e., DIANA-microT (34), ElMMo (35), MicroCosm (36), miRanda (37), miRDB (38), PicTar (39), and TargetScan (40). Interactions between marker miRNAs and mRNAs with low expression as established by RNA-seq were visualized using the R package circlize v. 0.4.15 (41). Functional gene enrichment analysis of identified miRNA-targeted mRNAs per microvascular compartment was performed using a similar statistical approach as aforementioned. The unadjusted *P* value obtained from differential expression analysis of RNA-seq data was used as a rank weight in the KS test.

### miRNA Quantitative RT-PCR

Total RNA from LMD samples and whole kidney samples was used for cDNA synthesis (*n* = 8). The miRCURY LNA RT kit (Qiagen, Hilden, Germany) was used for universal miRNA reverse transcription. The RNA input for reverse transcription was 4 µL for LMD samples and 10 ng for whole kidney samples. Reverse transcription was performed in 10 µL volumes in 0.2 mL 8-strip PCR Tubes Individually Attached Flat Caps Xtra-Clear (STARLAB, Hamburg, Germany) using an Eppendorf Mastercycler Nexus X2 Thermal Cycler (Eppendorf, Hamburg, Germany) with the following settings: 60 min at 42°C and 5 min at 95°C. A spike-in miRNA from *Caenorhabditis elegans* (cel-miR-39-3p) was added before reverse transcription and detected by quantitative PCR to assess cDNA synthesis efficiency. Samples that deviated >1 cycle from the mean cel-miR-39-3p spike-in quantification cycle (C_q_; formerly known as threshold cycle) (42) were discarded, and a new cDNA synthesis was performed for these samples.

cDNA was diluted 1:100 in 1× miRCURY LNA SYBR Green Master Mix (Qiagen), and 10 µL/well was added to custom miRCURY LNA miRNA PCR panel 96-well plates (Qiagen) to detect 24 selected miRNAs (Table 1). Plates were sealed with Xtra-Clear Advanced Polyolefin StarSeal (STARLAB). Quantitative PCR was performed with a LightCycler 96 System (Roche, Basel, Switzerland) according to the following cycling conditions: 2 min at 95°C and 45 two-step amplification cycles of 10 s at 95°C and 1 min at 56°C. Data were analyzed using LightCycler 96 software (v. 1.1.0.1320, Roche). If aberrant melting curves of quantitative PCR products were encountered, these samples were excluded from further analysis. miRNA values were obtained through C_q_ values, which were normalized to the geometric mean (GeoMean) of the three reference small RNAs RNU5G, SNORD68, and mmu-miR-16-5p. miRNA transcription levels relative to reference small RNAs were calculated by 2^−ΔCq^.

**Table 1.** miRCURY LNA-enhanced miRNA primers for quantitative RT-PCR-based detection of miRNA in mouse samples

Target	Assay ID
RNU5G	YP00203908
SNORD68	YP00203911
mmu-miR-16-5p	YP00205702
mmu-miR-126a-3p	YP00204227
mmu-miR-133a-3p	YP00204788
mmu-miR-143-3p	YP00205992
mmu-miR-145a-5p	YP00204483
mmu-miR-140-3p	YP00204304
mmu-miR-100-5p	YP00205689
mmu-miR-125b-5p	YP00205713
mmu-miR-322-3p	YP00205416
mmu-miR-351-5p	YP00205011
mmu-miR-23b-3p	YP02119314
mmu-miR-24-3p	YP00204260
mmu-miR-27b-3p	YP00205915
mmu-miR-652-3p	YP00204387
mmu-miR-6240	YP02103559
mmu-miR-2137	YP00205680
mmu-miR-5106	YP02109643
mmu-miR-200b-3p	YP00206071
mmu-miR-107-3p	YP00204468
mmu-miR-5119	YP02107854
mmu-miR-486a/b-5p	YP00204001
mmu-miR-451a	YP02119305

### mRNA Quantitative RT-PCR

Total RNA from LMD samples and whole kidney samples was used for cDNA synthesis (*n* = 8). cDNA was prepared from RNA (11.5 µL input) using random hexamers (Promega, Madison, WI), RNase OUT inhibitor (Invitrogen), and SuperScript III reverse transcriptase (Invitrogen) according to the manufacturer’s instructions. Reverse transcription was performed in 20-µL volumes in 0.2-mL MicroAmp 8-Tube Strip with Attached Domed Caps (Applied Biosystems, Waltham, MA) on a Biometra TProfessional standard 96 Thermocycler (Analytik Jena, Jena, Germany) with the following settings: 5 min at 25°C, 60 min at 50°C, and 15 min at 70°C.

cDNA of LMD samples (1 µL input) and whole kidney samples (5 ng input) was added to MicroAmp Optical 384-well plates (Applied Biosystems) and then evaporated with a SpeedVac Savant SPD1010 (Thermo Fisher Scientific, Waltham, MA) for 1 h at 45°C. Absolute qPCR ROX Mix (Thermo Fisher Scientific) and the appropriate Assay-on-Demand FAM-MGB-labeled hydrolysis probes (Applied Biosystems) (Table 2) were added to the plate at 10 µL/well. Plates were sealed using MicroAmp Optical Adhesive Film (Applied Biosystems). Quantitative PCR was performed on a ViiA 7 Real-Time PCR System (Applied Biosystems) according to the following cycling conditions: 15 min at 95°C and 50 two-step amplification cycles of 15 s at 95°C and 1 min at 60°C. Data were analyzed using QuantStudio Real-Time PCR software (v. 1.3, Applied Biosystems). mRNA values were obtained through C_q_ values, which were normalized to the mean of the reference genes *Gapdh* and peptidylprolyl isomerase A (*Ppia*) unless otherwise indicated. If no samples in a group yielded detectable C_q_ values, they were annotated as not detectable. mRNA transcription levels relative to reference genes were calculated by 2^−ΔCq^.

**Table 2.** Assay-on-demand primers* for quantitative RT-PCR-based detection of mRNA in mouse samples

Target	Assay ID
*Gapdh*	Mm99999915_g1
*Ppia*	Mm02342430_g1
*Pecam1*	Mm00476702_m1
*Plvap*	Mm00453379_m1
*Emcn*	Mm00497495_m1
*Ehd3*	Mm00517465_m1
*Cdh5*	Mm00486938_m1
*Kcnab1*	Mm00440018_m1
*Cytl1*	Mm01217843_m1
*Ptgis*	Mm00447271_m1
*Bmx*	Mm00515368_m1
*Vwf*	Mm00550376_m1
*Gabrb1*	Mm00433461_m1
*Adgrl3*	Mm01216694_m1
*Akr1b7*	Mm00477605_m1
*Rab3b*	Mm00772238_m1
*Rhpn1*	Mm00492435_m1
*Slc5a10*	Mm02600345_m1
*Pzp*	Mm00431533_m1
*Slc22a2*	Mm00457295_m1
*Cd36*	Mm00432403_m1
*Ace2*	Mm01159006_m1
*Igf1*	Mm00439560_m1
*Nd2*	Mm04225288_s1
*Nd1*	Mm04225274_s1

*Additional information required by minimum information for publication of quantitative real-time PCR experiments guidelines (42) is available upon request from the manufacturer.

### miRNA In Situ Hybridization

ISH was performed using miRNAscope HD Reagent kit RED (Advanced Cell Diagnostics, Newark, CA) on 10-µm-thick fresh-frozen mouse kidney sections according to the manufacturer’s instructions, with the exception of tissue fixation in 4% (wt/vol) paraformaldehyde (Sigma-Aldrich)-PBS (Thermo Fisher Scientific), which was extended to 2 h instead of the recommended 1 h. Selected miRNAs were detected using target-specific miRNAscope probes (Advanced Cell Diagnostics) (Table 3). For the detection of mmu-miR-140-3p, protease incubation was extended from 30 min to 45 min. Scrambled probes were consistently devoid of staining (Supplemental Fig. S1). Sections were scanned using the NanoZoomer 2.0-HT (Hamamatsu Photonics, Hamamatsu, Japan) to acquire digital images. Scale bars were added manually based on image magnification with information obtained using Aperio ImageScope (v. 12.3.3.5048, Leica).

**Table 3.** miRNAscope target probes for detection of miRNA via in situ hybridization in mouse samples

Target	Catalog Number
RNU6 (positive control)	727871-S1
Scrambled (negative control)	727881-S1
mmu-miR-126a-3p	728971-S1
mmu-miR-140-3p	1148971-S1
mmu-miR-322-3p	1147201-S1
mmu-miR-451a	1149501-S1

### Immunohistochemistry

Zinc-fixed paraffin-embedded mouse kidney sections were cut (5 µm) with an Epredia HM 355S microtome (Epredia, Portsmouth, NH) and baked in an oven at 60°C for 1 h. Sections were then deparaffinized in xylene followed by a rehydration series. Heat-induced epitope retrieval was performed using 10 mM Tris-1 mM EDTA (Sigma-Aldrich) buffer (pH 9.0), 1 mM EDTA buffer (pH 8.0), or 10 mM sodium citrate buffer (pH 6.0, Sigma-Aldrich) for 20 min. Fresh-frozen mouse kidney sections were cut (5 µm) at −20°C with a Leica CM1950 cryostat (Leica), followed by 10 min of acetone fixation at room temperature.

Endogenous peroxidases were blocked in both zinc-fixed paraffin-embedded and fresh-frozen sections with 0.0375% H_2_O_2_ (Merck, Kenilworth, NJ) in PBS for 20 min. Antibodies were diluted in 5% (vol/vol) FCS (ScienCell, Carlsbad, CA)-PBS or 1% (wt/vol) BSA (Sigma-Aldrich)-PBS (Table 4). Primary antibody was incubated for 1 h, followed by secondary antibody incubation for 45 min. Subsequently, EnVision+ System HRP-Labeled Polymer Anti-Rabbit (Agilent Technologies) was incubated for 30 min. Signal detection was performed with 3-amino-9-ethylcarbazole (Sigma-Aldrich) for 8 min, followed by counterstaining with Mayer’s hematoxylin. Isotype controls were consistently devoid of staining (Supplemental Fig. S2). Sections were washed thoroughly with PBS between every incubation step, and the entire protocol was performed at room temperature. Sections were mounted using Aquatex (Sigma-Aldrich) and scanned using the NanoZoomer 2.0-HT (Hamamatsu). Scale bars were added manually based on image magnification with information obtained using Aperio ImageScope (v. 12.3.3.5048, Leica).

**Table 4.** Antibodies for immunohistochemical protein detection in mouse samples

Target	Final Concentration	Diluent	Antigen Retrieval	Manufacturer	Catalog/Clone Number
PECAM1	0.07 µg/mL	5% FCS/PBS	Tris/EDTA (pH 9.0)	Cell Signaling	77699
PLVAP	NA	5% FCS/PBS	Tris/EDTA (pH 9.0)	Hybridoma supernatant	MECA-32
EMCN*	8.6 µg/mL	5% FCS/PBS	Tris/EDTA (pH 9.0)	NA	V.7C7.1
EHD3	2.4 µg/mL	5% FCS/PBS	Tris/EDTA (pH 9.0)	ProteinTech	25320-1-AP
vWF	0.07 IU	5% FCS/PBS	NA	Agilent Technologies	A0082
GABRB1	5.5 µg/mL	5% FCS/PBS	EDTA (pH 8.0)	Thermo Fisher Scientific	PA5-116439
ACE2	0.36 µg/mL	5% FCS/PBS	EDTA (pH 8.0)	Cell Signaling	92485
IGF1	2 µg/mL	1% BSA/PBS	Sodium citrate (pH 6.0)	Novus Biologicals	NBP2-48922
Secondary antibodies					
Rabbit anti-rat	3.33 µg/mL	5% FCS/PBS	NA	Vector Laboratories	AI-4001-.5
Isotype controls					
Rabbit IgG	Adapted to experimental conditions	Adapted to experimental conditions	Adapted to experimental conditions	Southern Biotech	0111-01
Rat IgG1				Antigenix	CN210020
Rat IgG2a				Antigenix	CN220020

NA, not applicable. *Kind gift from Prof. Dr. Dietmar Vestweber.

### Statistical Analysis

Statistical analysis of results was performed by one-way ANOVA. When the mean of each column was compared with the mean of every other column, Tukey’s post hoc test was used. When all column means were compared with a selected control column, Dunnett’s multiple comparison test was performed. When a limited number of selected column pairs was compared, Šidák’s multiple comparisons test was applied. If only two groups were present in a graph, an unpaired two-tailed *t* test was performed. Each group was required to have a minimum of three data points to be included in statistical testing. Statistical analyses were performed using GraphPad Prism (v. 9.4.0, GraphPad Software, San Diego, CA). All individual data points are presented in graphs, where bars depict means ± SD. Differences were considered statistically significant when *P* ≤ 0.05.

## RESULTS

### Combining LMD With (Small) RNA Sequencing

**Figure 1.**
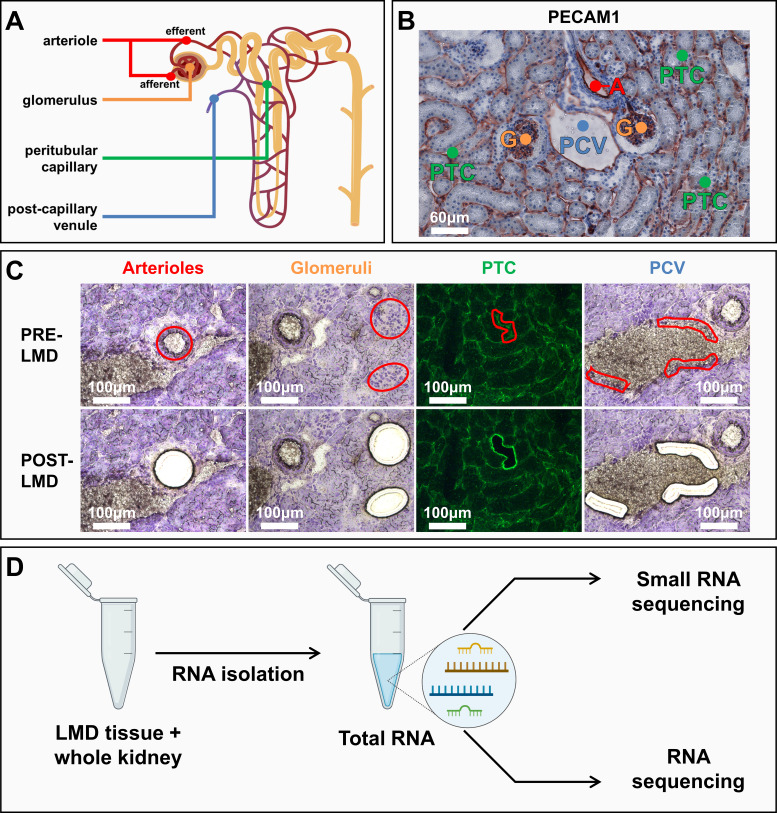
Schematic overview of the workflow for the investigation of mouse renal microvascular compartment-specific miRNA and mRNA transcription profiles in health. *A*: illustration of a kidney nephron indicating the four renal cortex microvascular compartments studied. *B*: immunohistochemical staining of the endothelial marker platelet/endothelial cell adhesion molecule 1 (PECAM1) identifying the four microvascular compartments of interest: arterioles (A), glomeruli (G), peritubular capillaries (PTC), and postcapillary venules (PCV). *C*: representative images of hematoxylin- and FITC-conjugated *Griffonia simplicifolia* lectin I isolectin B4-stained mouse kidney before (pre) and after (post) laser microdissection (LMD) of the microvascular beds (*n* = 8 mice). *D*: overview of the post-LMD workflow. Total RNA was isolated from LMD microvascular beds and whole kidney samples (*n* = 8 per sample type), followed by two separate library preparations to enable small RNA sequencing and RNA sequencing (*n* = 4 per sample type). Images in *A* and *D* were created with BioRender.com.

In the present study, we aimed to establish the molecular basis that underlies endothelial heterogeneity in different microvascular compartments in the mouse kidney cortex through a new experimental design that combined LMD with small RNA-seq and RNA-seq (Fig. 1, *A* and *B*). To study microvascular heterogeneity at the miRNA and mRNA level in the healthy mouse kidney, we used LMD to collect arterioles, glomeruli, peritubular capillaries, and postcapillary venules (Fig. 1*C*). This technique allowed for selection of microvascular segments based on their histological appearances in the kidney. LMD samples, alongside whole kidney samples, were processed for small RNA-seq and RNA-seq (Fig. 1*D*).

**Figure 2.**
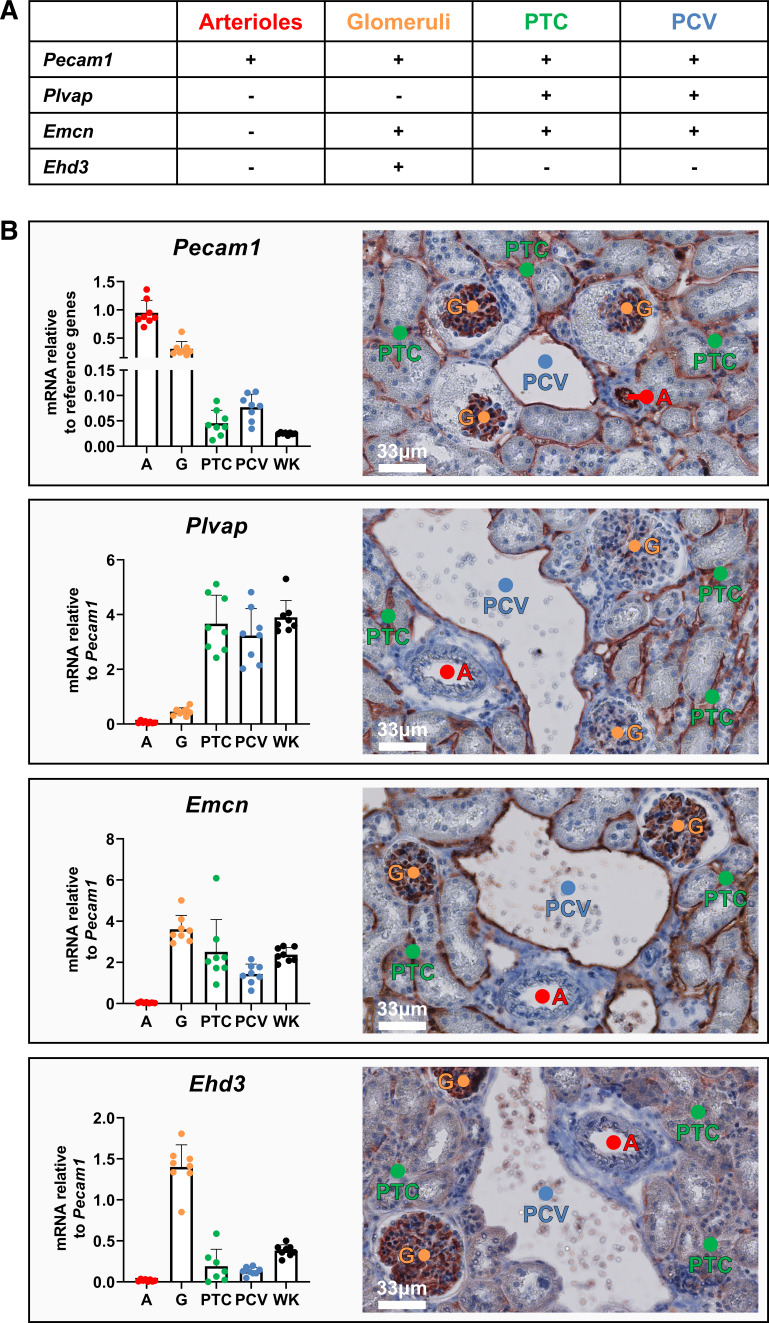
Confirmation of quality of histology-based laser microdissection (LMD) of mouse kidney cortex microvascular compartments. *A*: expected gene transcription levels per microvascular compartment based on the literature. *B*: gene transcription levels in LMD samples and whole kidney (WK) samples as determined by quantitative RT-PCR (*n* = 8; *left*) and detection of corresponding protein expression patterns (*n* = 3; *right*) in the kidneys of healthy mice. Quantitative RT-PCR data were normalized to the mean quantification cycle (C_q_) of *Gapdh* and peptidylprolyl isomerase A (*Ppia*; for *Pecam1*) or *Pecam1* (for *Plvap*, *Emcn*, and *Ehd3*). Statistical testing was performed using one-way ANOVA. For *Pecam1*, LMD samples were compared with the WK: *P* < 0.0001 [for arterioles (A) and glomeruli (G)], *P* = 0.9943 (for PTC), and *P* = 0.8499 (for PCV). For *Plvap*, arterioles and glomeruli were compared with PTC and PCV: *P* < 0.0001 (all tested comparisons). For *Emcn*, arterioles were compared with glomeruli, PTC, and PCV: *P* < 0.0001 (arterioles vs. glomeruli and PTC) and *P* = 0.0072 (arterioles vs. PCV). For *Ehd3*, glomeruli were compared with arterioles, PTC, and PCV: *P* < 0.0001 (all tested comparisons). +, high expression; −, low/no expression; *Ehd3*, EH domain containing 3; *Emcn*, endomucin; PCV, postcapillary venules; *Pecam1*, platelet/endothelial cell adhesion molecule 1; *Plvap*, plasmalemma vesicle-associated protein; PTC, peritubular capillaries.

Prior to (small) RNA-seq, we confirmed that the microvascular segments isolated by LMD were properly enriched by determining mRNA levels of genes with known expression patterns in the microvascular compartments. For this purpose, we selected platelet/EC adhesion molecule 1 (*Pecam1*), plasmalemma vesicle-associated protein (*Plvap*), endomucin (*Emcn*), and EH domain containing 3 (*Ehd3*), the expected mRNA transcription patterns of which were based on previously established protein expression patterns, as shown in Fig. 2*A* (10–12, 15). The pan endothelial marker *Pecam1* was detected in all microvascular compartments. *Pecam1* mRNA transcription was highest in glomeruli and arterioles, indicating either relatively high endothelial content in these compartments or higher *Pecam1* transcription per EC (Fig. 2*B*). *Plvap* mRNA transcription was in line with its protein expression, which showed the highest expression levels in peritubular capillaries and postcapillary venules, whereas *Emcn* mRNA exhibited low transcription in arterioles, and *Ehd3* mRNA was almost exclusively transcribed in glomeruli (Fig. 2*B*). Microvascular gene expression patterns were similar when cadherin 5 (*Cdh5*) was used as a reference gene instead of *Pecam1* (data not shown). Statistical comparisons of gene transcription levels are provided in Supplemental Fig. S3*A*. Analysis of endothelial genes in LMD cortical tubules also revealed significant endothelial enrichment in peritubular capillaries relative to tubules (Supplemental Fig. S3, *B* and *C*). Together, these data indicate that microvascular beds were accurately compartmentalized with LMD and that microvascular compartments retained their unique gene transcription patterns following LMD.

### Microvascular Compartments Exhibit Unique miRNA Profiles

**Figure 3.**
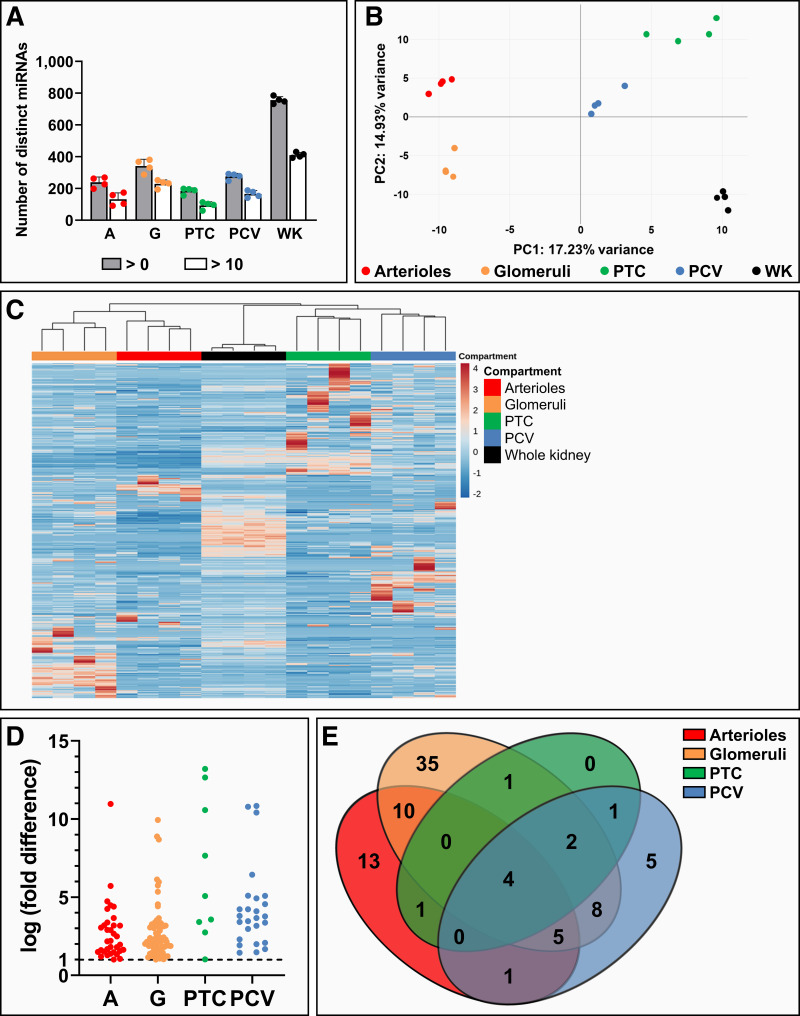
Small RNA sequencing of microvascular segments of the healthy mouse kidney revealed microvascular compartment-specific miRNome profiles. Small RNA sequencing was performed on healthy mouse kidney arterioles (A), glomeruli (G), peritubular capillaries (PTC), postcapillary venules (PCV), and whole kidney (WK) samples (*n* = 4). *A*: number of unique miRNAs detected per sample (gray bars) and unique miRNAs detected >10 times per sample (white bars). *B*: clustering of samples with similar miRNA transcription profiles by principal component analysis. *C*: heatmap visualizing relative transcription levels of all detected miRNAs based on unsupervised hierarchical clustering, revealing microvascular compartment-specific miRNA transcription profiles. *D*: differential expression analysis uncovered enriched miRNAs per microvascular segment relative to the WK. Each data point represents a unique miRNA with a false discovery rate < 0.05 and log (fold difference) > 1. *E*: Venn diagram visualized both compartment-specific and shared miRNA transcription between microvascular compartments.

To study miRNA transcription patterns in microvascular compartments, small RNA-seq was performed on microvascular compartments. The whole kidney was included as a reference group. In microvascular samples, 200–400 unique miRNAs were detected, whereas 700–800 unique miRNAs were found in the whole kidney (Fig. 3*A*). Principal component analysis showed a grouping pattern based on microvascular compartment, indicating the existence of microvascular compartment-specific miRNA transcription profiles (Fig. 3*B*), a finding that was affirmed by unsupervised hierarchical clustering (Fig. 3*C*).

To identify miRNAs with enriched transcription in microvascular compartments, differential expression analysis compared with the whole kidney was performed (Fig. 3*D* and Supplemental Fig. S4). Enriched miRNAs were found in all four compartments, with the number of enriched miRNAs observed being lowest in peritubular capillaries and highest in glomeruli. Several miRNAs appeared to be uniquely enriched in a single microvascular compartment, yet various miRNAs were also shared between two or more compartments (Fig. 3*E*). A full list of miRNAs that were included in the Venn diagram is provided in Supplemental Table S1.

### Identification and Validation of Compartment-Specific Marker miRNAs

**Figure 4.**
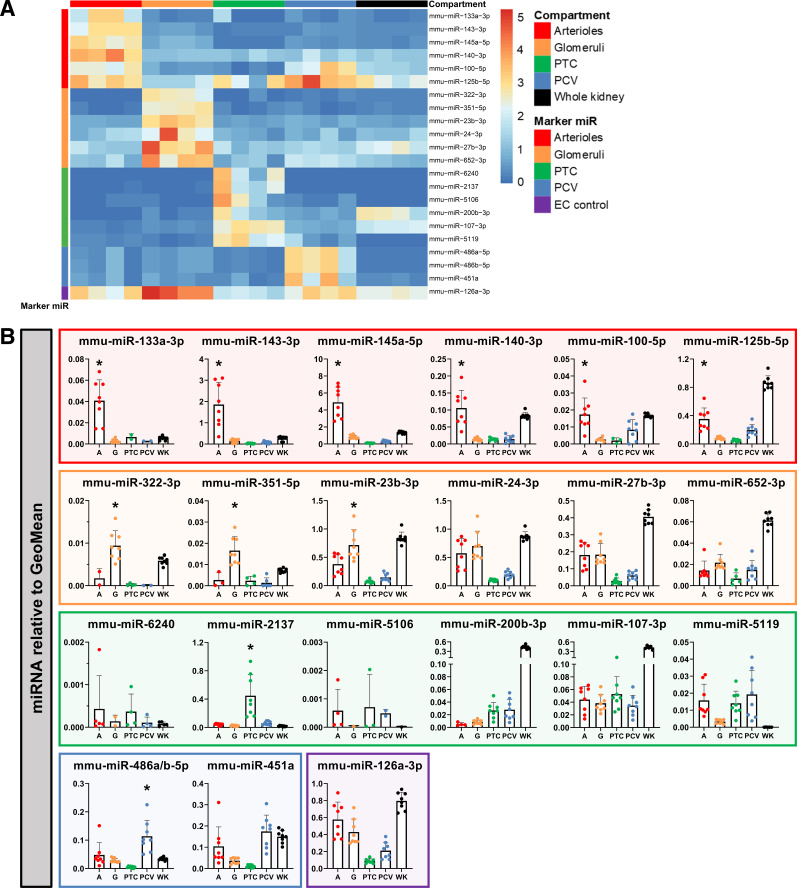
Identification and validation of microvascular compartment-specific marker miRNAs. Marker miRNAs were defined as miRNAs with higher transcription in a single microvascular compartment compared with transcription in the other microvascular compartments. *A*: heatmap showing the top six marker miRNAs per microvascular compartment as identified with small RNA sequencing. The false discovery rate was <0.05 for all included miRNAs. mmu-miR-126a-3p was included as a reference for endothelial input. *B*: all miRNAs shown in the heatmap were validated by quantitative RT-PCR (*n* = 8) in arterioles (A), glomeruli (G), peritubular capillaries (PTC), postcapillary venules (PCV), and whole kidney (WK) samples. Color-bordered squares indicate marker miRNAs for arterioles (red), glomeruli (orange), PTC (green), PCV (blue), and the endothelial cell (EC) control (purple). Data were normalized to the GeoMean of RNU5G, SNORD68, and mmu-miR-16-5p. Statistical testing was performed using one-way ANOVA. Significant enrichment (*P* ≤ 0.05) of marker miRNAs over the other microvascular compartments is indicated by *. The complete statistical analysis is available in Supplemental Fig. S5.

“Marker miRNAs” are miRNAs that exhibit significantly higher transcription in one microvascular compartment compared with the others. Relative transcription of the top six enriched marker miRNAs for each microvascular compartment is shown in Fig. 4*A.* Endothelium-enriched mmu-miR-126a-3p was included as an endothelial reference, as it is transcribed in all ECs. Quantitative RT-PCR confirmed compartment-enriched transcription patterns of the majority of miRNAs identified based on the small RNA-seq data set (Fig. 4*B*). All six arteriolar marker miRNAs showed significant enrichment in arterioles with quantitative RT-PCR. For the glomerular compartment, significant enrichment compared with the other microvascular compartments was detected for mmu-miR-322-3p, mmu-miR-351-5p, and mmu-miR-23b-3p. In peritubular capillaries, mmu-miR-2137 was the only miRNA shown by quantitative RT-PCR to have enriched transcription. The other candidates identified by small RNA-seq were either not reliably detected or showed comparable levels of transcription in at least one other microvascular compartment. Significant enrichment of postcapillary venule marker miRNAs was found for mmu-miR-486a/b-5p but not for mmu-miR-451a, which showed comparable transcription levels between postcapillary venules and arterioles. The results of the multiple comparison tests are provided in Supplemental Fig. S5.

**Figure 5.**
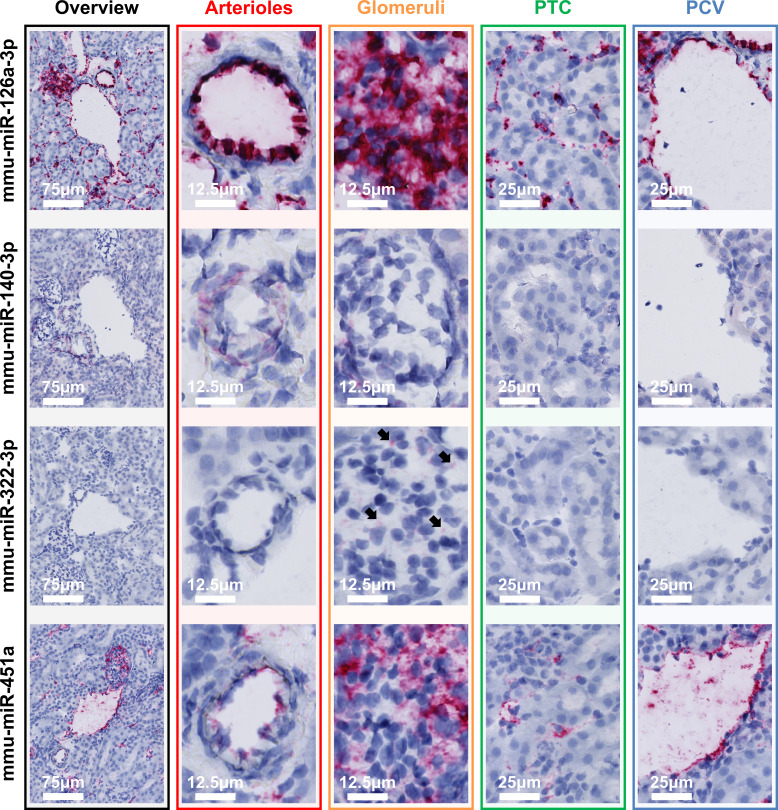
In vivo localization of microvascular compartment-specific marker miRNAs. Localization of mmu-miR-126a-3p, mmu-miR-140-3p, mmu-miR-322-3p, and mmu-miR-451a in fresh-frozen kidney tissue from healthy mice using miRNA in situ hybridization is shown. Per miRNA, images representative of four mice are shown. PCV, postcapillary venules; PTC, peritubular capillaries.

Next, a selection of miRNAs was visualized by ISH to investigate their localization pattern in the kidney (Fig. 5). ECs in all microvascular compartments transcribed mmu-miR-126a-3p, with the signal intensity being highest in arterioles and glomeruli. mmu-miR-140-3p, indicated as an arteriolar marker miRNA by small RNA-seq and quantitative RT-PCR, was confirmed by ISH to be detected in arterioles. Small regions exhibiting a positive mmu-miR-140-3p signal were also frequently detected immediately adjacent to glomeruli, coinciding with the expected location of afferent and efferent arterioles. For mmu-miR-322-3p, a glomerular marker miRNA, a positive signal was exclusively detected in glomeruli, although the overall intensity of the signal was low. mmu-miR-451a localized in the endothelial lining of venules and was also present in the other microvascular compartments, with signal intensity appearing highest in glomeruli and postcapillary venules.

### Microvascular Compartments Have Distinct Gene Transcription Profiles

**Figure 6.**
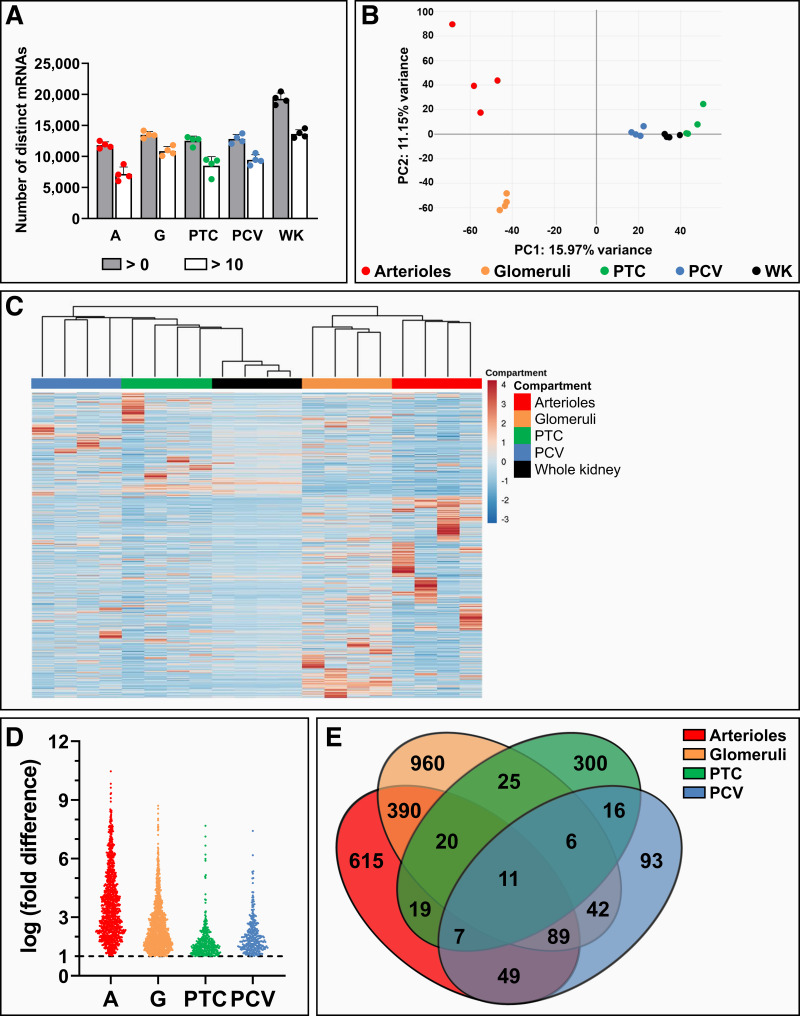
RNA sequencing of microvascular segments of the healthy mouse kidney revealed microvascular compartment-specific transcriptome profiles. RNA sequencing was performed on healthy mouse kidney arterioles (A), glomeruli (G), peritubular capillaries (PTC), postcapillary venules (PCV), and whole kidney (WK) samples (*n* = 4). *A*: number of unique mRNAs detected per sample (gray bars) and unique mRNAs detected >10 times per sample (white bars). *B*: clusters of samples with similar gene transcription profiles visualized by principal component analysis. *C*: heatmap visualizing relative transcription levels of all detected mRNAs based on unsupervised hierarchical clustering, revealing microvascular compartment-specific mRNA transcription profiles. *D*: differential expression analysis uncovered enriched gene transcription per microvascular segment relative to the WK. Each data point represents a unique mRNA with a false discovery rate < 0.05 and log (fold difference) > 1. *E*: Venn diagram visualized both compartment-specific and shared mRNA transcription between microvascular compartments.

RNA-seq of arterioles, glomeruli, peritubular capillaries, and postcapillary venules was performed to further characterize their transcriptomes, with the whole kidney being included as a reference. The number of unique genes detected varied between 11,000 and 15,000, with no large differences in gene count between microvascular compartments (Fig. 6*A*). Grouping of samples in the principal component analysis plot was based on compartment origin (Fig. 6*B*), which was confirmed by unsupervised hierarchical clustering (Fig. 6*C*), indicating that each microvascular compartment exhibited a unique gene transcription profile.

Genes with enriched transcription compared with the whole kidney were identified in all microvascular compartments, with the number of enriched genes ranging from 300 in postcapillary venules to 1,500 in glomeruli (Fig. 6*D* and Supplemental Fig. S6). Over 70% of enriched genes were microvascular compartment specific (Fig. 6*E*). A full list of mRNAs that were included in the Venn diagram is provided in Supplemental Table S2.

### Identification and Validation of Compartment-Specific Marker mRNAs

**Figure 7.**
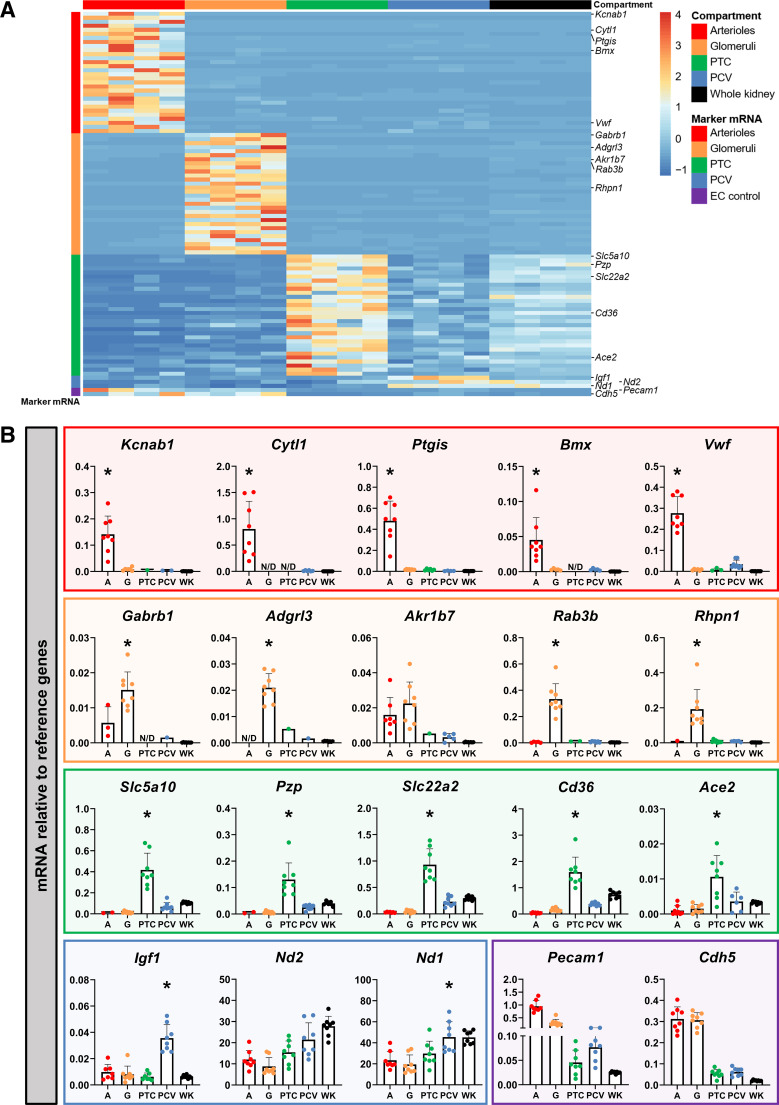
Identification and validation of microvascular compartment-specific marker mRNAs. Marker mRNAs were defined as mRNAs with higher transcription in a single microvascular compartment compared with transcription in the other microvascular compartments. *A*: heatmap showing the top 30 marker mRNAs per microvascular compartment as identified with RNA sequencing. The false discovery rate was <0.05 for all included mRNAs. Platelet/endothelial cell adhesion molecule 1 (*Pecam1*) and cadherin 5 (*Cdh5*) were included as a reference for endothelial input. The identity of all included marker mRNAs is provided in Supplemental Table S3. *B*: a selection of genes present in the heatmap was validated in arterioles (A), glomeruli (G), peritubular capillaries (PTC), postcapillary venules (PCV), and whole kidney (WK) samples by quantitative RT-PCR (*n* = 8). Color-bordered squares indicate marker mRNAs for arterioles (red), glomeruli (orange), PTC (green), PCV (blue), and the endothelial cell (EC) control (purple). Data were normalized to the average quantification cycle (C_q_) of *Gapdh* and peptidylprolyl isomerase A (*Ppia*). Statistical testing was performed using one-way ANOVA. Significant enrichment (*P* ≤ 0.05) of marker mRNAs over the other microvascular compartments, excluding other microvascular compartments without detectable levels (N/D), is indicated by *. The complete statistical analysis is available in Supplemental Fig. S7.

To map endothelial heterogeneity at the mRNA transcription level, we identified marker mRNAs with significantly higher transcription in a single microvascular compartment compared with the others. The number of marker mRNAs identified through RNA-seq ranged from 512 in arterioles to 3 in postcapillary venules, with the top 30 enriched genes shown in Fig. 7*A.* The identity of the top 30 marker mRNAs is provided in Supplemental Table S3. A selection of these genes was validated using quantitative RT-PCR (Fig. 7*B* and Supplemental Fig. S7). The quantitative RT-PCR data reproduced the compartment-enriched transcription pattern that was uncovered with RNA-seq for all marker mRNAs (Fig. 7*B*). The only exception was the putative glomerular marker mRNA aldo-keto reductase family 1 member B7 (*Akr1b7*), which showed no differences between arterioles and glomeruli and was therefore not successfully validated as glomerular marker mRNA.

**Figure 8.**
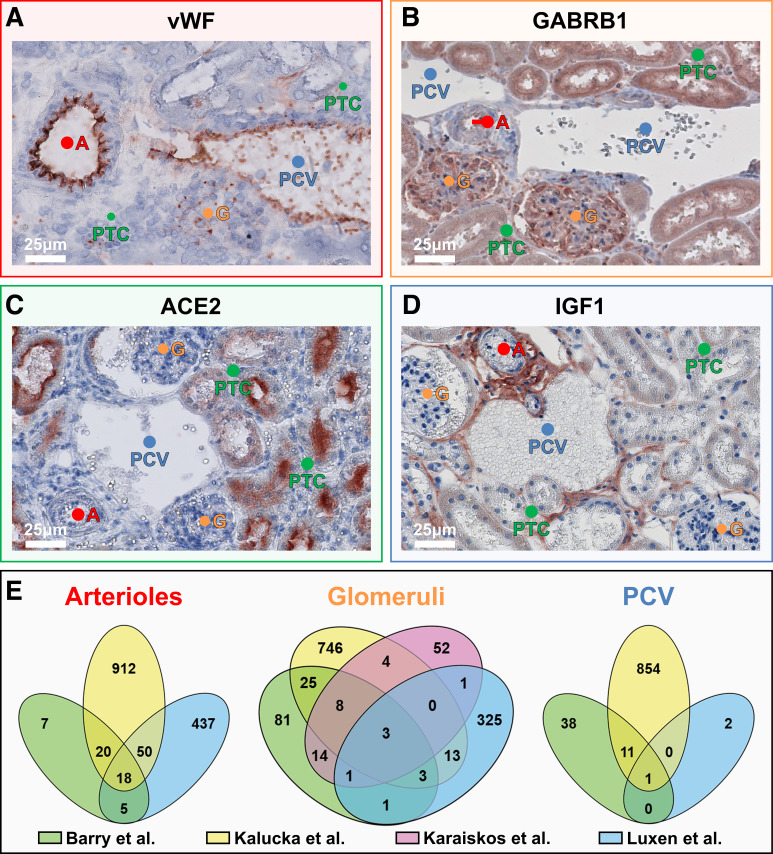
Microvascular compartment-specific marker mRNA expression. *A–D*: localization of von Willebrand factor (vWF; *A*), γ-aminobutyric acid type A receptor subunit β1 (GABRB1; *B*), angiotensin-converting enzyme 2 (ACE2; *C*), and insulin-like growth factor 1 (IGF1; *D*) protein in mouse kidney tissue using immunohistochemical detection. Per protein, images representative of three mice are shown. Locations of arterioles (A), glomeruli (G), peritubular capillaries (PTC), and postcapillary venules (PCV) are indicated. Color-bordered squares indicate predicted enriched expression in arterioles (red), glomeruli (orange), PTC (green), and PCV (blue). Immunohistochemistry was performed using zinc-fixed paraffin-embedded tissue except for vWF, which was performed on fresh-frozen tissue. *E*: marker genes per microvascular compartment as established via laser microdissection/RNA sequencing (RNA-seq) (Luxen et al., present study) were compared with marker genes identified in endothelial cell (EC) single-cell RNA-seq studies [Barry et al. (15), Kalucka et al. (14), and Karaiskos et al. (16) for arterioles, glomeruli, and PCV]. The Karaiskos et al. (16) data set only included glomerular ECs. A full list of mRNAs that were included in the Venn diagrams is provided in Supplemental Table S4.

Next, we used IHC to assess whether protein expression of marker mRNAs showed similar microvascular compartment-specific expression patterns. IHC of the arteriole marker von Willebrand factor confirmed protein expression in the endothelial lining of arterioles and, to a lesser extent, in venous ECs (Fig. 8*A*). The glomerular marker γ-aminobutyric acid type A receptor subunit β1 (GABRB1) showed expression in glomeruli and, other than a sporadic presence in peritubular capillaries, was not found in other microvascular compartments, which was consistent with the mRNA data (Fig. 8*B*). For angiotensin-converting enzyme 2 (ACE2) protein, only a signal was found in the tubular lumen, but not in peritubular capillaries (Fig. 8*C*). The postcapillary venule marker insulin-like growth factor 1 (IGF1) showed a positive signal in the endothelial lining of venules and also showed a scattered presence in peritubular capillaries. No IGF1 protein signal was detected in the endothelial lining of arterioles and glomeruli (Fig. 8*D*).

*Igf1* enrichment in venous renal ECs was also reported by EC scRNA-seq studies (14, 15). This prompted a more detailed analysis of overlap between microvascular endothelial gene enrichment identified by combining LMD and RNA-seq and the genes reported by three EC scRNA-seq studies (14–16). In arterioles and glomeruli, considerable overlap was found between enriched genes identified with either method (Fig. 8*E*). For postcapillary venules, *Igf1* was the only gene shared by all studies, although our analysis merely revealed three postcapillary venule-enriched genes, thus preventing a more extensive comparison. No comparison between LMD/RNA-seq and scRNA-seq could be provided for marker genes in peritubular capillaries, as this was not a defined EC subset in the included scRNA-seq studies. Notably, the EC scRNA-seq studies all identified a large number of genes that were not shared by any other study, indicating that scRNA-seq studies experienced considerable interstudy variation despite shared methodologies.

### Microvascular Compartment-Specific miRNA-mRNA Pairs

**Figure 9.**
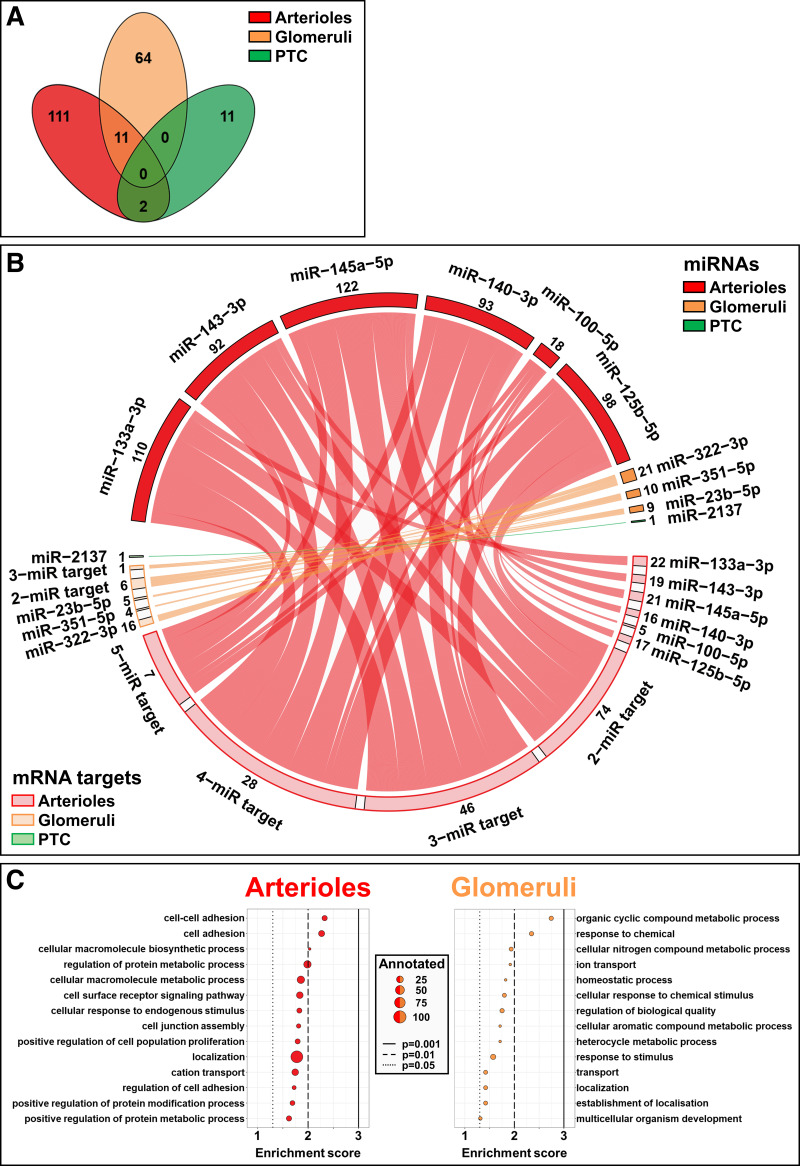
Identity and predicted function of microvascular compartment-specific miRNA-mRNA pairs. *A*: pathway enrichment analysis was performed for all enriched genes per microvascular compartment. The overlap between enriched pathways in arterioles, glomeruli, and peritubular capillaries (PTC) is shown. A complete list of enriched pathways is available in Supplemental Table S5. *B*: circos plot showing connections between microvascular compartment-specific miRNAs and predicted gene targets that exhibit significantly lower microvascular mRNA expression levels in our laser microdissection/RNA-sequencing dataset. Threads represent connections between marker miRNAs (top half) and their predicted mRNA targets (bottom half), and thread color denotes different microvascular compartments. mRNAs grouped in the “*n*-miR target” clusters are targeted by multiple miRNAs, with different combinations of miRNAs included in the same cluster. A complete list of the identified miRNA-mRNA pairs is available in Supplemental Table S6. *C*: the top 14 enriched pathways of all mRNAs predicted to be targeted by marker miRNAs in *B* for arterioles and glomeruli. miRNA, microRNA.

To determine the biological processes per microvascular compartment in which genes with enriched transcription were involved, we performed functional pathway enrichment analysis. We identified 199 significantly enriched pathways in total, the majority of which were exclusive to a single compartment (Fig. 9*A*). In arterioles, we predominantly found biological processes related to muscle contraction and cytoskeleton organization, whereas glomeruli represented a more diverse collection of processes, and in peritubular capillaries, enrichment of various metabolic processes was identified (Supplemental Table S5).

Next, we investigated whether we could integrate our miRNA and mRNA datasets to identify unique miRNA-mRNA pairs with putative roles in microvascular compartment function. As miRNAs repress translation of their target mRNAs, we assessed the overlap between predicted target mRNAs of validated marker miRNAs and mRNAs that exhibited significantly lower microvascular expression. In this manner, we identified 533 miRNA-mRNA pairs in arterioles, 40 pairs in glomeruli, and 1 pair in peritubular capillaries (Fig. 9*B*). We then performed functional pathway enrichment analysis for arterioles and glomeruli to predict the biological processes in which the identified miRNA-targeted mRNAs partake. For arterioles, we identified pathways related to cell adhesion, whereas in glomeruli we found various metabolic processes, thereby providing a first insight into biological processes potentially regulated by miRNAs in renal microvascular compartments (Fig. 9*C*).

## DISCUSSION

In this study, we successfully combined LMD of renal microvascular compartments with small RNA-seq and RNA-seq to characterize microvascular heterogeneity. Using this approach, we uncovered distinct miRNome and transcriptome profiles of arterioles, glomeruli, peritubular capillaries, and postcapillary venules. In addition, we identified multiple marker miRNAs and mRNAs that exhibited enriched transcription in a single microvascular compartment and uncovered microvascular compartment-specific miRNA-mRNA pairs, which potentially underlie functional microvascular heterogeneity. These findings highlight the importance of compartmentalizing complex tissues into smaller components before making molecular maps, as whole organ analysis resulted in loss of spatial information and concealment of microvascular compartment-specific signatures.

The combination of LMD with high-throughput screening is not without precedent (21), but this is the first time (small) RNA-seq has been applied to LMD microvascular compartments. Following these analyses, we selected a single miRNA per compartment to investigate its compartment enrichment by ISH. Since we focused on microvascular ECs, we selected mmu-miR-140-3p as an arteriole marker miRNA, because mmu-miR-133a-3p, mmu-miR-143-3p, and mmu-miR-145a-5p were previously described to be highly transcribed in vascular smooth muscle cells (43–45). The present results indicated that mmu-miR-140-3p is predominantly transcribed in the smooth muscle layer of arterioles.

The glomerular marker miRNA mmu-miR-322-3p showed a compartment-exclusive transcription pattern, as it was consistently detected in every glomerulus and absent from all other renal structures. Although mmu-miR-322-3p is not preserved in humans, it does share more than 70% homology with human hsa-miR-424-3p. hsa-miR-424-3p is described to play important roles in endothelial-to-mesenchymal transition and angiogenesis (46, 47). Whether mmu-miR-322-3p is involved in similar processes in mice remains to be investigated.

In peritubular capillaries, mmu-miR-2137 exhibited strongly enriched transcription over the other microvascular compartments, as shown with quantitative RT-PCR. Proof of its localization being restricted to peritubular capillaries could, however, not be provided due to a lack of specific probes. The same applied to the postcapillary venule marker miRNA mmu-miR-486a/b-5p, so mmu-miR-451a was selected for ISH instead. mmu-miR-451a was detected in all microvascular compartments and was present in the endothelial lining of venules and glomeruli and, to a lesser extent, in arterioles and peritubular capillaries. A positive mmu-miR-451a signal was also detected in the lumen of larger blood vessels. This latter signal likely originated from erythrocytes, which also contain high levels of mmu-miR-451a (48). Since mmu-miR-451a has not been reported before to be associated with EC function, it is at present not possible to provide more information on its putative role in the microvasculature of the mouse kidney cortex.

Various marker miRNAs have been described in the context of renal diseases. For instance, decreased plasma levels of the postcapillary venule marker miR-451a were correlated with diminished estimated glomerular filtration rate, a kidney function parameter, in patients undergoing coronary angiography (49). In a mouse endotoxemia model, mmu-miR-451a transcription was significantly increased in the kidney after 24 h (50). The arteriole marker miRNA miR-140-3p was shown to be downregulated in renal biopsies of patients with chronic kidney disease with end-stage renal disease (51). In addition, miR-140-3p was significantly decreased in a rat model of diabetic nephropathy, with a proposed role in the onset and progression of the disease (52). Further research is required to confirm whether these described changes in miRNA transcription levels in blood or kidney tissue are associated with altered renal microvascular behavior. Identifying the source of these miRNAs will reveal whether the identified microvascular compartment-enriched marker miRNAs can be used as plasma and/or urine biomarkers to indicate early endothelial dysfunction in specific microvascular compartments.

In addition to miRNAs, we also identified mRNAs with microvascular compartment-enriched transcription patterns. For instance, *Gabrb1* and adhesion G protein-coupled receptor L3 (*Adgrl3*) transcription were glomerular enriched relative to the other microvascular compartments. Both genes code for cell surface receptor proteins that are predominantly described in a neuronal context (53, 54). Interestingly, various genes that were initially considered to be neuron exclusive are also transcribed by glomerular podocytes (55). GABRB1 protein expression showed a glomerular-enriched pattern, yet the observed protein expression pattern was distinct from that of glomerular ECs detected by, e.g., PECAM1 and EHD3 staining. Localization of GABRB1 in podocytes is highly likely considering its reported podocyte-enriched expression in a glomerular single-cell atlas (16).

IGF1 protein is predominantly produced by liver hepatocytes and secreted into the blood (56). Our mRNA data indicated enriched transcription of this gene in venules compared with other renal microvascular compartments, which confirmed venous EC expression in scRNA-seq studies (14, 15). IHC showed noncontinuous expression of IGF1 protein in the endothelial lining of postcapillary venules, with alternating IGF1-positive and IGF1-negative areas. Scattered expression of IGF1 was detected in peritubular capillaries, and the extracellular matrix surrounding arterioles and postcapillary venules also showed a strong positive signal for IGF1. This is likely due to IGF-binding proteins being present in the extracellular matrix, which can act as a reservoir for IGF1 (57, 58).

The use of LMD inevitably leads to the generation of samples that also include vascular support cells and minor contamination with other renal structures. Apart from successfully collecting resident ECs as shown in Fig. 2, we also identified in all studied microvascular compartments genes transcribed by these support cells, e.g., smooth muscle cell-specific genes in arterioles and podocyte-exclusive genes in glomeruli. The endothelial lining of peritubular capillaries and postcapillary venules represents a thin layer that is closely associated with bordering tubular epithelial structures. Consequently, some level of contamination with tubular material following LMD of these microvascular beds could not be avoided. This also explains why our analysis revealed enriched transcription of *Ace2* mRNA in peritubular capillaries compared with the other microvascular beds, whereas immunohistochemical protein detection indicated that ACE2 was exclusively expressed in tubules. This contamination by nonendothelial structures necessitates techniques that visualize their location, such as ISH and/or IHC. At the same time, the presence of vascular support cells in LMD samples also presents opportunities to identify enriched ligand-receptor pairs in specific microvascular beds to increase understanding of local cell-cell communication. Novel technologies such as Digital Spatial Profiling also exploit the advantage of retaining spatial information, yet studies on their application to further delineate microvascular heterogeneity are still awaited (59).

In the past years, scRNA-seq has led to an increased appreciation of the sheer number of endothelial subpopulations present in an organ (13–17). As both scRNA-seq and our LMD/RNA-seq approach encompass advantages and disadvantages, we were interested in comparing enriched gene expression between EC subsets established via scRNA-seq and the genes identified in the present study. Substantial overlap in microvascular-enriched genes existed between LMD/RNA-seq and scRNA-seq studies. However, this comparison also revealed that many genes were not shared between studies, neither between scRNA-seq and LMD/RNA-seq nor between different scRNA-seq data sets. Although discrepancies between LMD/RNA-seq data and scRNA-seq data can be explained by the presence of vascular support cells in the former method, scRNA-seq enzymatic cell dissociation protocols may have introduced artifacts associated with drift in gene expression (18). Together, these data identify overlapping genes with likely enriched microvascular EC expression patterns, yet the numerous differences between studies also highlight the variability in outcomes regardless of the technique used. Another limitation of these studies, ours included, is that only male animals were included. Sex-based differences in baseline gene transcription levels have already been described in ECs (60), and it would be of great interest to assess whether the here described microvascular compartment-specific m(i)RNA transcription patterns are shared with those in females or differ in a sex-related manner.

miRNA-mRNA pair analysis revealed more than 550 microvascular compartment-specific connections, which may contribute to heterogeneous microvascular gene and protein expression patterns. Most miRNA-mRNA pairs were identified in arterioles, suggesting that miRNA-based gene repression may play a more central role here compared with other microvascular compartments. A striking example of the implications of miRNA-based target repression is EMCN, which in the kidney was highly expressed by all ECs except arteriolar ECs (11). miRNA-mRNA pair analysis revealed that *Emcn* is targeted by four miRNAs with high abundance in arterioles; hence, it is likely that miRNA-dependent repression of *Emcn* is responsible for the absence of EMCN protein expression by ECs in arterioles, although further studies will need to confirm this. Furthermore, functional pathway enrichment analysis of miRNA-targeted genes in arterioles revealed their involvement in cell adhesion-related processes. Leukocyte recruitment in the kidney was reported to take place in capillaries and postcapillary venules, but not in arterioles (61–63). The lack of leukocyte recruitment in arterioles may therefore be partially due to miRNA-based repression of the required cellular machinery, thereby further demonstrating functional implications of miRNA-induced gene repression.

The present study focused on differences in miRNA and mRNA transcription profiles between microvascular compartments in health. The same workflow as described here can now be applied to investigate transcription profiles in renal diseases. We hypothesize that the here uncovered molecular microvascular heterogeneity has impact on local responses to, e.g., proinflammatory stimuli. Using the current setup of combining LMD-based sample generation with sequencing platforms creates a highly sensitive approach to study such differential responses between microvascular beds.

### Perspectives and Significance

Here, we presented a combination of two techniques that together successfully identified microvascular compartment-specific miRNA and mRNA signatures in the mouse kidney cortex. Although the biological functions of validated microvascular compartment-specific marker miRNAs and mRNAs are at present largely unknown, we provided a first glance by performing functional pathway enrichment analyses of compartment-specific miRNA-mRNA pairs. The experimental setup presented in this study provides a firm basis for follow-up studies into renal microvascular heterogeneity in disease conditions.

## DATA AVAILABILITY

The sequencing data discussed in this report have been deposited in the National Center for Biotechnology Information's Gene Expression Omnibus (GEO) and are accessible through GEO Series Accession No GSE220987 (https://www.ncbi.nlm.nih.gov/geo/query/acc.cgi?acc=GSE220987).

All other raw data underlying the figures shown in this report are accessible through Figshare: https://doi.org/10.6084/m9.figshare.21593376.

## SUPPLEMENTAL DATA

10.6084/m9.figshare.21895842 Supplemental Figs. S1−S7 and Supplemental Tables S1−S6: https://doi.org/10.6084/m9.figshare.21895842.

## GRANTS

This project is cofinanced by the Ministry of Economic Affairs and Climate Policy by means of the PPP allowance made available by Top Sector Life Sciences & Health to stimulate public-private partnerships (No. 6334, to M.v.M. and G.M.). This work was also supported by a grant awarded by the Foundation De Cock-Hadders (No. 2020-55, to M.L.).

## DISCLOSURES

M. Hackl is co-founder and CEO/CSO of TAmiRNA, which provides small RNA sequencing and RNA sequencing analysis services. A. B. Diendorfer, M. Pultar, and S. Skalicky are employed by TAmiRNA. G. Molema is co-founder and CSO/CTO of Vivomicx, which provides laser microdissection analysis services. Vivomicx and TAmiRNA financially contributed to the PPP allowance. None of the other authors have any conflicts of interest, financial or otherwise, to disclose.

## AUTHOR CONTRIBUTIONS

M.L., M.v.M., and G.M. conceived and designed research; M.L., P.J.Z., F.M., R.M.J., T.K., J.M., S.S., and A.B.D. performed experiments; M.L., P.J.Z., F.M., R.M.J., M.P., S.S., and A.B.D. analyzed data; M.L., M.H., M.v.M., and G.M. interpreted results of experiments; M.L. and M.P. prepared figures; M.L., M.v.M, and G.M. drafted manuscript; M.L., P.J.Z., F.M., R.M.J., T.K., J.M., M.P., S.S., A.B.D., M.H., M.v.M, and G.M. edited and revised manuscript; M.L., P.J.Z., F.M., R.M.J., T.K., J.M., M.P., S.S., A.B.D., M.H., M.v.M., and G.M. approved final version of manuscript.
